# Diagnostic Potential of a Recombinant *Candida albicans* Hyr1 Protein

**DOI:** 10.1007/s11046-025-01025-6

**Published:** 2025-11-14

**Authors:** Marta Bregón-Villahoz, Ander Díez, Jon Galech, Maria-Soledad Cuétara, Giulia Carrano, Maria-Dolores Moragues, Iñigo Fernandez-de-Larrinoa, Inés Arrieta-Aguirre

**Affiliations:** 1 Department of Immunology, Microbiology and Parasitology, University of the Basque Country EHU, Barrio Sarriena s/n, 48940 Leioa, Bizkaia Spain; 2 Department of Nursing I, University of the Basque Country EHU, Leioa, Bizkaia Spain; 3 Severo Ochoa University Hospital, Leganés, Madrid Spain; 4 Department of Applied Chemistry, University of the Basque Country EHU, San Sebastian, Gipuzkoa Spain

**Keywords:** Hyr1, CAGTA, ELISA, Invasive candidiasis, Diagnosis, *Pichia pastoris*

## Abstract

Invasive candidiasis (IC) is a life-threatening fungal infection caused by *Candida* species. Current diagnostic methods are based on blood culture of the fungus, a technique with limited sensitivity and slow turnaround times. To address these limitations, novel diagnostic strategies are under investigation. This study evaluates the diagnostic potential of the *Candida albicans* germ tube protein Hyr1 and a subterminal Hyr1 fragment (D22b), both produced in an eukaryotic expression system, for the diagnosis of IC; for that purpose, recombinant Hyr1 and D22b were expressed in *Pichia pastoris* and tested by ELISA using sera from 176 patients at risk of invasive fungal infections. The diagnostic performance of these antigens was determined and compared with other biomarkers (CAGTA and β-D-glucan). Interestingly, the recombinant proteins exhibited higher apparent molecular weights than predicted, suggesting the presence of post-translational modifications. Serological detection of antibodies against the recombinant Hyr1 and D22b fragment successfully distinguished patients with IC caused by the most commonly isolated *Candida* species, achieving sensitivities greater than 70% and specificities above 80%. These findings highlight the potential of the serological detection of antibodies to Hyr1 and D22b as a promising diagnostic approach that overcomes the drawbacks of CAGTA detection and could serve as a valuable complement to blood culture, supporting earlier diagnosis and guiding timely treatment decisions in IC. Furthermore, comparing results obtained with antigens produced in eukaryotic and prokaryotic systems, results suggest that accurate protein folding and post-translational processing influence the success of the diagnostic technique.

## Introduction

Invasive candidiasis (IC) is the most prevalent among the hospital acquired fungal infections [1], especially in the intensive care unit, where it is estimated that one third of *Candida* bloodstream infections occur [2], with an associated mortality that ranges between 40 and 55% [3].

The prognosis of IC is strongly associated with early diagnosis and correct adherence to treatment [4]. However, diagnosis is challenging as, most of the time, clinical manifestations are nonspecific and similar to some severe bacterial infections [5]. For this reason, empirical therapy is often used in high-risk patients [6], but this application is controversial [7] and has associated economic costs. Diagnosis is mainly based on observation of the fungus, either by microscope direct examination or by growth on a culture medium. The current gold standard for IC diagnosis, blood culture, has a sensitivity of 50% for the full spectrum of these infections, detecting most cases of candidemia, while the detection decreases significantly in deep-seated candidiasis, especially when not associated with candidemia [8]. An additional limitation of this technique is the time required to test positive, as it can take up to 96 h, so results would not be available until late stages of the infection [9]. For these reasons, new culture-independent tests are needed for the early detection of all presentations of the infection in order to anticipate specific antifungal treatment. Furthermore, appropriate diagnostic techniques to rapidly exclude IC in high-risk patients would allow to de-escalate or discontinue antifungal treatment, reducing healthcare costs and the risk of inducing possible resistance to these drugs.

The detection of CAGTA (*Candida albicans* germ tube antibodies) is a technique developed by our group based on the detection of antibodies against the mycelial phase of *Candida* *albicans* [10] thus allowing discriminating colonisation from invasion. Several studies have proved the efficacy of the CAGTA titre determination as diagnostic and prognostic tool even though its sensitivity and specificity are moderate [11, 12]; however, it should be noted that it is a subjective and non-automatable method. The Invasive Candidiasis (CAGTA) VirClia Monotest® (Vircell Microbiologists, Granada, Spain) [13] has been recently developed and appears to provide a rapid and fully automated assay; even though it is a promising tool for IC diagnosis, evidence supporting its clinical utility is still limited, and it requires specific equipment that may not be available in all laboratories. In order to address the mentioned limitations, in a previous study [14], we screened a library of bacteriophages expressing *C.* *albicans* proteins and identified the Hyphally regulated cell wall protein 1 (Hyr1) as one of the CAGTA targets and, more specifically, the target epitopes appeared to be located in a subterminal region of the Hyr1 protein, within the region we call D22b. The fact that CAGTA specifically recognize the whole protein and its specific region supports their immunogenic role and points to both Hyr1 and D22b as clinically relevant targets for serological detection of IC.

In this study, we developed an enzyme-linked immunosorbent assay (ELISA) to evaluate the detection of specific antibodies against Hyr1 and the D22b fragment as biomarkers for diagnosing patients at risk of developing an invasive fungal infection (IFI), to this end, we used an eukaryotic expression system. In addition, the data were compared with the results of (1–3)-β-D-glucan (BDG) determination and CAGTA assay to assess the diagnostic utility of these serum biomarkers.

## Materials and methods

### Coding sequences for protein expression

DNA of *Candida albicans* SC5314 was used as a template to amplify the Hyr1 protein gene, from nucleotides 61 to 2,685 (taking as reference the NCBI sequence XM_717090.2), and the D22b region that comprises nucleotides 1,441 to 2,011. A pair of primers was designed for each target, containing an additional “caca” sequence and the sequences for restriction sites: 5’ CACAGGCCCAGCCGGCCTTAGAAGTTGTCACAAGCAG 3’ (Hyr1 Forward Primer, Tm = 50.2 °C), 5’ CACAGCGGCCGCATTTTCATTAGTATCAATAGTTGGAAC 3’ (Hyr1 Reverse Primer, Tm = 50.1 °C), 5’ CACAGAATTCGGTCAATCTACTATTTACGTCAACCTG 3’ (D22b Forward Primer, Tm = 55.2 °C) and 5’ CACAGCGGCCGCTTCATTTGAACCAGAACCACCTTCAGAACCT 3’ (D22b Reverse Primer, Tm = 61.4 °C).

PCRs were performed in a GeneAmp® PCR System 9700 thermocycler (Applied Biosystems, Massachusetts, USA) using the Phusion™ High-Fidelity DNA Polymerase (Thermo Fisher Scientific, Vilnius, Lithuania) and 25 µM of each primer. The PCR program was set up with an initial denaturation step at 98 °C for 30 s, followed by 30 cycles of denaturation at 98 °C for 10 s, primer annealing for 30 s (at 58.8 °C for Hyr1 and at 64.2 °C for D22b amplification) and extension at 72 °C for 150 s for Hyr1 and 30 s for D22b. The program ended with an elongation step at 72 °C for 10 min.

### Cloning and expression in *Pichia pastoris*

The target sequences were cloned into the yeast expression vector pPICZα™ (Invitrogen, California, USA) that provides a secretion signal and a C-terminal 6 × histidine tag for the recombinant proteins. Hyr1 coding sequence was cloned into the pPICZαB vector between the restriction sites of the enzymes SfiI and NotI (Thermo Fisher Scientific, Massachusetts, USA), while the D22b fragment was cloned into the pPICZαA vector between the restriction sites of EcoRI and NotI (Thermo Fisher Scientific, Massachusetts, USA). Digested plasmids and amplicons were ligated with the T4 DNA ligase (Thermo Fisher Scientific, Massachusetts, USA).

The recombinant plasmids were used to transform *Escherichia coli* Subcloning Efficiency DH5α™ (Invitrogen, California, USA) using the water heat-shock method (15 min), and transformants were selected based on Zeocin™ (25 μg/mL; Invitrogen, California, USA) resistance on low salt Lysogeny Broth agar (LB-Lennox; Condalab, Madrid, Spain) plates and subsequently sequenced. After plasmid linearization with SacI endonuclease, *Pichia pastoris* X-33 (Invitrogen, California, USA) competent cells were transformed following the protocol of the EasyComp™ kit provided in the EasySelected™ *Pichia* Expression kit (Invitrogen, California, USA). The positive transformants were selected through culture on Yeast extract Peptone Dextrose agar (YPD agar; 1% yeast extract, 2% peptone, 2% agar (Conda, Madrid, Spain), 2% dextrose (Panreac, Barcelona, Spain)) with 100 mg/ml Zeocin. The selected yeast clones were cultured for protein expression. A single colony of each transformation was inoculated in Buffered glycerol- complex medium (BMGY; 1.34% Yeast Nitrogen Base (YNB) with ammonium sulphate and without amino acids (BD, New Jersey, USA), 4 × 10^–5^% biotin (Sigma Aldrich, Missouri, USA), 1% glycerol (Panreac, Barcelona, Spain) in 100 mM potassium phosphate buffer pH 6.0) and incubated at 30 °C and 270 rpm up to OD_600_ = 2–6, then cells were collected and suspended at OD_600_ = 1 in Buffered methanol-complex medium (BMMY; 1.34% Yeast Nitrogen Base (YNB) with ammonium sulphate and without amino acids (BD, New Jersey, USA), 4 × 10^–5^% biotin (Sigma Aldrich, Missouri, USA), 0.5% methanol (Panreac, Barcelona, Spain) in 100 mM potassium phosphate buffer pH 6.0) and reincubated, adding methanol to a final concentration of 0.5% every 24 h.

Recombinant proteins production was assessed by SDS-PAGE followed by Coomassie staining and Western blotting revealed with a specific 6x-His Tag® antibody linked to Horseradish peroxidase (GT359 clone; ab184607, Abcam, Amsterdam, Netherlands). Proteins were purified from culture supernatants by Nickel-affinity chromatography with HisTrap™ Excel Prepacked Columns (GE HealthCare, Uppsala, Sweden) and the Äkta Start Protein Purification System (GE HealthCare, Uppsala, Sweden). The eluted proteins were dialyzed against PBS with Slide-Lyzer™ MINI Dialysis Devices, 10 K MWCO (Thermo Fisher Scientific, Illinois, USA) and protein concentration was estimated with the Bradford Protein Reagent (Thermo Fisher Scientific, Illinois, USA).

### Sera sample collection

Sera from 176 patients were collected prospectively and stored at − 20 °C. These sera are part of an anonymised sera collection registered at the Instituto de Salud Carlos III (C.0005025).

Patients were classified into three groups according to clinical and microbiological data. Group I included 86 patients with proven invasive candidiasis by a positive *Candida* sp. blood culture or biopsy (Table 1). Group II gathered 47 patients with invasive fungal infections (IFIs) caused by non- *Candida* species (Table 1). Group III included 43 patients with no evidence of IFI. If more than one serum from the same patient was available in the collection, the serum closest to the date of positive culture (for Group I patients), or those with high or positive CAGTA or BDG titres, were selected (for Group II and III patients). These sera are part of an anonymised sera collection registered at the Instituto de Salud Carlos III (C.005025). CAGTA titre and BDG values were retrieved from a previous study of our group that evaluated the same sera [15]. Briefly, CAGTA titre was determined following Moragues et al. [16] with modifications. Serum diluted in PBS was incubated with *C. albicans* blastospores and then, serial dilutions of the supernatant were incubated with fixed *C. albicans* germ tubes. Following washing and incubation with FITC-conjugated anti-Human IgG antibodies, samples were examined under a fluorescence microscope. Positive results showed green fluorescence on germ tubes. On the other hand, Serum BDG was estimated with the Fungitell kit (Assoc. Cape Cod, USA), following the manufacturer instructions.

**Table 1:** Distribution of fungal species isolated in groups I and II

	Microorganism	Number of patients
Group I	*Candida albicans*	41
	*Candida glabrata* (currently *Nakaseomyces glabratus)*	16
	*Candida parapsilosis*	12
	*Candida tropicalis*	4
	*Candida krusei* (currently *Pichia kudriavzevii*)	3
	*Candida lusitaniae*	2
	*Candida guilliermondii* (currently *Meyerozyma guilliermondii*)	2
	*Candida* sp.	1
	Mixed *Candida* infection	5
Group II	*Aspergillus* spp.	26
	*Pneumocystis* spp.	14
	*Scedosporium* spp.	3
	*Conidiobolus* sp.	1
	*Purpureocillium* sp.	1
	*Cryptococcus* sp.	1
	Zygomycota	1

### Determination of serum antibodies against Hyr1 and D22b by Enzyme-Linked ImmunoSorbent Assay (ELISA)

The presence of antibodies to the specific protein/fragment of interest in human sera was assessed by indirect ELISA as described by Díez et al. [15]. Optimal assay conditions for each protein were established in a checkerboard assay. Absorbance at 492 nm was measured using an Infinite 50R microplate reader (Tecan, Grödig, Austria) and registered with the software Magellan™ F50 (Tecan, Grödig, Austria).

In order to normalize the results of different day assays, we run the same control serum in every plate as an internal reference and results were reported as relative absorbance values to this reference serum.

Mean values of relative absorbance of the three groups of patients were compared by ANOVA test using IBM SPSS (version 28.0.2.2) and GraphPad Prism (version 8.2.1) softwares, and values of *p* < 0.05 were considered statistically significant. Receiver-operating-characteristic (ROC) curve analysis and area under the curve (AUC) value were used to evaluate the overall diagnostic performance of the tests, using the optimal cut-off values determined by the Youden index (Y). The diagnostic values of the assays were calculated as described by Kozinn et al. [17].

## Results

### Heterologous protein production

The recombinant proteins were produced in *P. pastoris* and then purified by affinity chromatography with the histidine tag. The SDS-PAGE analysis of aliquots obtained at different times during induction revealed the optimal point for Hyr1 expression at 36 h while that of the D22b fragment was 24 h.

Both recombinant products showed an apparent molecular weight near 3 times higher than expected according to their amino acid sequence, which could be due to the presence of posttranslational modifications. The analysis of these posttranslational modifications with the GPP tool [18] revealed 34 potential N-glycosylation and up to 224 O-glycosylation sites in the whole protein taking as a reference the UniProt Q5AL03 Hyr1 sequence, being most of them located in the subterminal region of the protein, specifically 11 N-glycosilation and 61 O-glycosylation sites in the D22b region.

### Estimation of serum anti-Hyr1 and anti-D22b antibodies by ELISA

The optimal assay conditions for each ELISA were determined by a checkerboard titration test. Serum dilution was set at 1/400 while the commercial secondary antibody was diluted 1/1,000 for the experiments. These conditions were selected as they provided the highest specific signal with minimal background in the test. Optimal concentration of antigen to be immobilised on the well surfaces was 0.034 µg/ml for the recombinant Hyr1 and 0.423 µg/ml for the recombinant D22b.

**Fig. 1:**
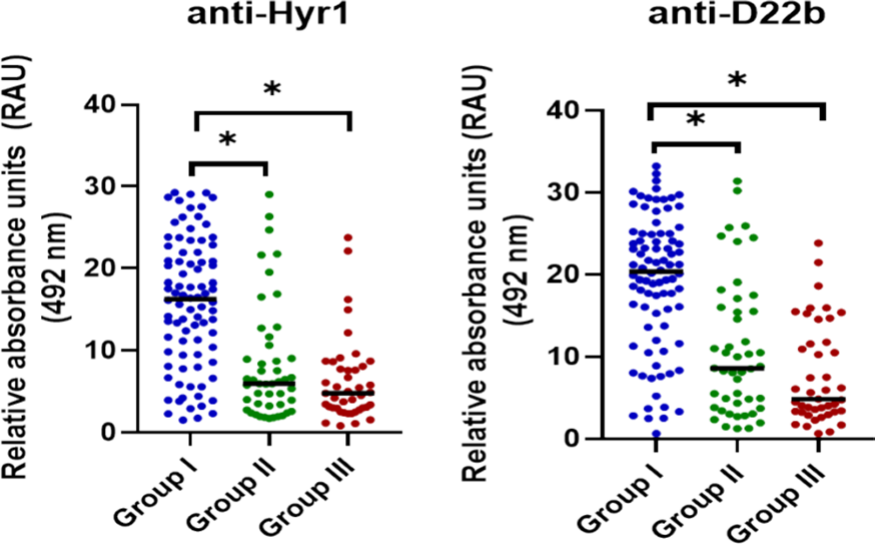
Distribution of human serum antibodies concentration (relative absorbance units; RAU) against recombinant Hyr1 and D22b by ELISA, in three groups of patients: group I) patients diagnosed with invasive candidiasis; group II) diagnosed with other invasive fungal infections; and group III) patients with no evidence of fungal infections. *Statistical significance in the concentrations of antibodies in the serum between the different patient groups *p* < 0.05

The results of the detection of antibodies against recombinant Hyr1 and D22b for the three groups of patients are shown in Fig. 1. For both antigens, the mean values of serum concentration of specific antibodies for patients with proven IC (group I) were higher than those of the two control groups (II and III) and these differences were statistically significant. In contrast, no significant differences were found for the mean values of groups II and III.

### Optimal cut-off point and ROC curves

**Fig. 2:**
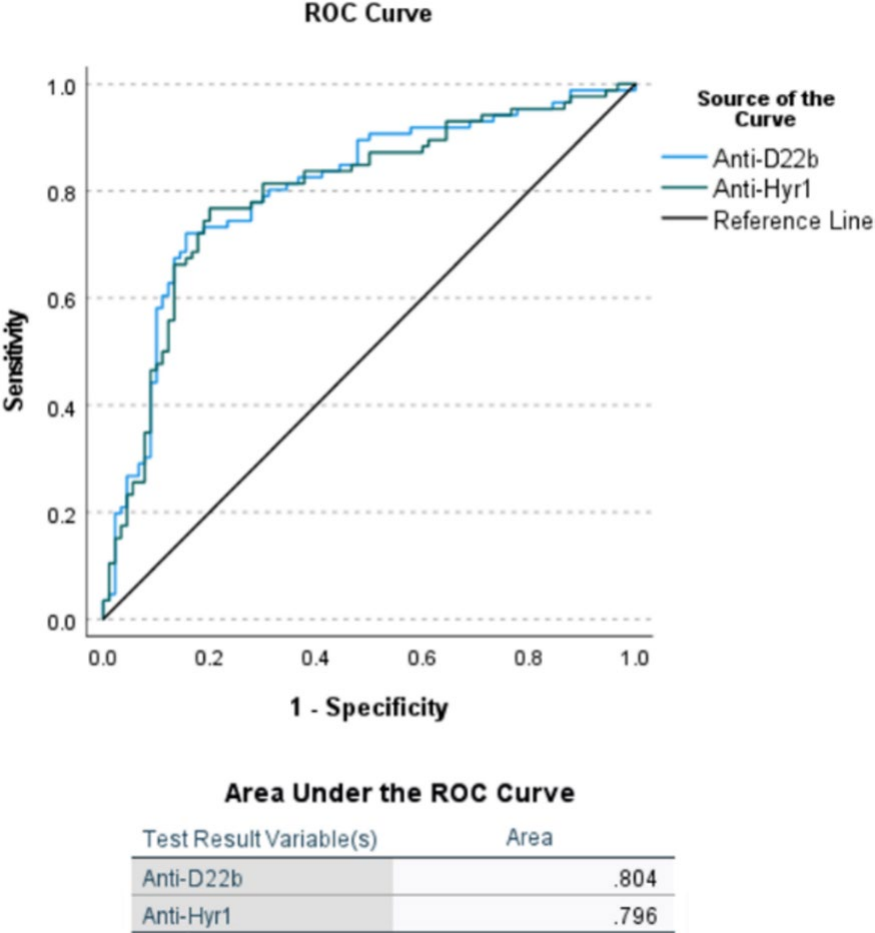
ROC (Receiver-operating-characteristic) curves and AUC (area under the curve) values for the anti-D22b and anti-Hyr1 ELISA tests. Reference line = no discrimination line

The performance of the Hyr1 and D22b antibody tests for the diagnosis of IC was determined through the analysis of the ROC curves (Fig. 2) with an optimal cut-off for the anti-Hyr1 ELISA of 9.2 (Y = 0.567) and 16.05 (Y = 0.565) for the anti-D22b test. The area under the curve (AUC) values for the detection of anti-D22b (0.804, 95% CI: 0.737–0.871) antibodies was slightly higher than that of anti-Hyr1 (0.796, CI: 0.728- 0.865), but this difference was not statistically significant.

### Diagnostic usefulness and comparison with CAGTA titration and BDG detection

After establishing the optimal cut-off point for both tests, the diagnostic parameters were calculated (Table 2). Data for CAGTA and BDG tests previously published [15] for this pool of sera have been included for comparison.

**Table 2:** Diagnostic parameters of the detection of antibodies against recombinant Hyr1 and D22b through ELISA, BDG and anti-germ tube antibodies titre determination (CAGTA)

	SE (%)	SP (%)	PPV (%)	NPV (%)	A (%)
Anti-Hyr1 (95% CI)	76.7 (67.8–85.7)	80.0 (71.7–88.3)	78.6 (69.8- 87.3)	78.3 (69.8–86.7)	78.4 (72.3–84.5)
Anti-D22b (95% CI)	72.1 (62.6–81.6)	84.4 (77.0–91.9)	81.6 (72.9–90.3)	76.0 (67.6–84.4)	78.4 (72.3–84.5)
BDG^a^ (95% CI)	60.5 (50.1–70.8)	57.8 (47.6–68.0)	57.8 (47.6–68.0)	60.5 (50.1–70.8)	59.1 (51.8–66.4)
CAGTA (80)^a^ (95% CI)	60.5 (50.1–70.8)	97.8 (94.7- 100)	96.3 (91.3–100)	72.1 (64.2–80.1)	79.5 (73.6–85.5)
CAGTA (160)^a^ (95% CI)	45.3 (34.8–55.9)	100 (100–100)	100 (100–100)	65.7 (57.7–73.6)	73.3 (66.8–79.8)
Anti-D22b + Anti-Hyr1 (95% CI)	80.2 (71.8–88.6)	77.8 (69.2–86.4)	77.5 (68.9–86.2)	80.5 (72.1–88.8)	79.0 (73.0–85.0)
CAGTA (160) + Anti-Hyr1 (95% CI)	80.2 (71.8–88.6)	80.0 (71.7–88.3)	79.3 (70.8–87.8)	80.9 (72.7–89.1)	80.1 (74.2–86.0)
CAGTA (160) + Anti-D22b (95% CI)	77.9 (69.1–86.7)	84.4 (77.0–91.9)	82.7 (74.5–91.0)	80.0 (72.0–88.0)	81.3 (75.5- 87.0)
CAGTA (80) + Anti-Hyr1 (95% CI)	82.6 (74.5–90.6)	78.9 (70.5–87.3)	78.9 (70.5–87.3)	82.6 (74.5–90.6)	80.7 (74.8–86.5)
CAGTA (80) + Anti-D22b (95% CI)	79.1 (70.5–87.7)	83.3 (75.6–91.0)	81.9 (73.6–90.2)	80.6 (72.6–88.7)	81.3 (75.5–87.0)
Anti-Hyr1 + BDG (95% CI)	88.4 (81.6–95.1)	45.6 (35.3–55.8)	60.8 (52.2–69.4)	80.4 (69.5–91.3)	66.5 (59.5- 73.5)
Anti-D22b + BDG (95% CI)	88.4 (81.6–95.1)	48.9 (38.6–59.2)	62.3 (53.7–70.9)	81.5 (71.1–91.8)	68.2 (61.3–75.1)

Abbreviations: CI = confidence interval; BDG = β-D-glucan; CAGTA (80) = *Candida albicans* germ tube antibodies detection with a 1/80 cut-off; CAGTA (160) = *Candida albicans* germ tube antibodies detection with 1/160 cut-off; SE = Sensitivity; SP = Specificity; PPV = Positive predictive value; NPV = Negative predictive value; A: accuracy

^a^Raw sera data from Diez et al. [15] for comparison

The detection of antibodies against Hyr1 was more sensitive compared with the detection of antibodies against D22b that was more specific, though, both assays had the same accuracy values. Both ELISAs were more sensitive than CAGTA but at the expense of reducing specificity. The same phenomenon was observed for the predictive values, where the ELISA tests had better NPVs, while the PPVs were better for the detection of CAGTA antibodies. In terms of accuracy, only the detection of CAGTA with a 1/80 cut-off surpassed that of the anti-Hyr1 and anti-D22b ELISA tests. In contrast, BDG assay reached the same sensitivity as CAGTA (80); however, BDG diagnostic parameters were much lower, as expected for a panfungal marker.

When compared to individual assays, the combination of the results increased the sensitivity and negative predictive value, while specificity and positive predictive value were either reduced or remained unchanged. The highest diagnostic accuracy was achieved by combining the detection results of anti-D22b antibodies with those of CAGTA detection, regardless of the applied cut-off.

A re-calculation of diagnostic sensitivity parameters of the tests was performed according to the species responsible for IC in group I patients (Table 3).

**Table 3:** Sensitivity of the diagnostic tests according to the *Candida* species responsible for the infection in group I patients

	Total	Anti- Hyr1		Anti- D22b		BDG		CAGTA (80)		CAGTA (160)	
	N	N	%	N	%	N	%	N	%	N	%
*C. albicans*	41	33	**80**	29	**71**	30	73	23	56	23	56
*C. glabrata*	16	13	**81**	13	**81**	9	56	13	81	8	50
*C. parapsilosis*	12	11	**92**	10	**83**	5	42	8	67	3	25
*C. tropicalis*	4	2	50	3	75	2	50	2	50	1	25
*C. krusei*	3	1	33	1	33	2	67	1	33	0	0
*C. guilliermondii*	2	0	0	0	0	0	0	0	0	0	0
*C. lusitaniae*	2	2	100	2	100	1	50	1	50	1	50
*Candida* sp.	1	0	0	0	0	0	0	0	0	0	0
Mixed infection	5	4	80	4	80	3	60	4	80	3	60

Abbreviations: BDG = β-D-glucan; CAGTA (80) = *Candida albicans* germ tube antibodies detection with a 1/80 cut-off; CAGTA (160) = *Candida albicans* germ tube antibodies detection with 1/160 cut-off

Only *C. albicans*, *Candida glabrata* and *Candida parapsilosis* were enough represented to draw conclusions. As a general rule, regarding these three species, Hyr1 and D22b ELISA tests proved to be more sensitive than BDG or CAGTA determinations. In addition, anti-Hyr1 ELISA resulted more sensitive than anti-D22b determination in the case of *C. albicans* and *C. parapsilosis* infections. Nevertheless, the differences in antibody response of patients infected with those species were not statistically significant for both ELISAs.

## Discussion

Invasive candidiasis (IC) affects approximately 750,000 people worldwide each year [19], with mortality rates exceeding 40%, depending on the type of infection and causative species [9, 20]. Diagnosis of these infections is complicated and relies mainly on blood culture, a technique that takes days to provide results and has a sensitivity of less than 50% for the whole spectrum of IC [7, 21].

Detection of BDG, a major component of the cell wall of most fungi, is the most widely used culture-independent method for the diagnosis of invasive fungal infections and the only one approved for use in both Europe and the USA [22, 23]. This method provides results within two hours and it can test positive 5–8 days before blood culture [24]; however, although it is a good indicator of invasive fungal infection, being a panfungal marker, it cannot provide a specific diagnosis of *Candida* infections [25].

Another diagnostic obstacle is the presence of *Candida* spp. as normal commensals in the human body. Alternative diagnostic tools are therefore being developed to detect biomarkers related to the invasive form of these microorganisms, such as hyphal formation in *C.* *albicans* [26]. In this sense, our research group developed an indirect immunofluorescence protocol for the detection of antibodies directed against antigens located on the surface of the germ tubes of *C. albicans* (CAGTA). CAGTA detection has proved useful for the diagnosis of IC [10, 16, 27, 28], but it is a non-automated technique whose interpretation is subjective, requiring the operator’s training and experience. The limitations of this technique could be avoided by developing ELISA tests to detect antibodies to antigens recognized by CAGTA. Regarding the cost-effectiveness of ELISA tests, this technique represents a reliable and efficient approach for sample analysis, as it requires minimal reagent volumes while allowing the simultaneous processing of a large number of specimens. Furthermore, the necessary equipment for its implementation is widely available in most clinical laboratories [29].

Several authors have proposed Hyr1, one of the proteins recognised by CAGTA, as a potential biomarker for IC [30, 31]. As far as we know, there is only one study, carried out in our group [32], using a recombinant Hyr1 protein produced in *Escherichia coli*, which reported high specificity (82.2%) but moderate sensitivity (58.3%) for the detection of anti-Hyr1 antibodies in a limited group of 36 IC patients and 45 negative control patients. In the current study, we obtained higher sensitivity values (76.7%) and still high specificity (80%). These differences could be due to the expression system, as bacterial expression systems usually limit proper folding of large eukaryotic proteins and post-translational modifications [33]. We used a yeast expression model and the heterologous proteins showed higher apparent molecular weights suggesting the presence of post-translational modifications, such as O- and N- glycosylations. The higher sensitivity seen in our data may be attributable to antigen conformations that closely mirror the native protein. It is also worth noting that we analysed twice as many patient sera as Laín and coworkers, which strengthens our results.

In addition to studying the whole Hyr1 protein, as some immunoprotection studies showed cross-reactivity of the N-terminal end of Hyr1 [34, 35] we decided to evaluate D22b, a sub-terminal Hyr1 segment. The detection of antibodies against the D22b fragment slightly increased the specificity of the test (84.4%) but decreased the sensitivity (72.1%) compared to the results obtained with the whole protein, although the accuracy was the same (78.4%) for both ELISA tests.

The results of our ELISA tests are comparable to those for the detection of antibodies against enolase or mannans, two of the most immunogenic *Candida* antigens investigated. The reported sensitivities and specificities for the detection of antibodies to Eno1 ranged from 70 to 88%, and from 83.9 to 94% respectively [36–38]. On the other hand, a meta-analysis [39] established a sensitivity and specificity for the detection of anti-mannan antibodies of 59% and 86%, respectively, although a recent study [40] reported a sensitivity of 74.6%.

Regarding the detection of antibodies against other *C. albicans* proteins, He and collaborators [37] studied the diagnostic value of serological responses to phosphoglycerate kinase (Pgk1) and β-(1,3) glycosyltransferase (Bgl2), obtaining high sensitivity (86.5 and 80.8%) and specificity (92 and 90%) results. However, their study was performed on 52 IC patients and 50 healthy individuals, without consideration of cross-reactivity with other fungal infections, as we have done. Sáez-Rosón and collaborators [41] also identified Pgk1, together with the methionine synthase (Met6), as CAGTA targets; in this line, our group reported good diagnostic values for the detection of anti-Met6 antibodies [15], especially in immunocompromised patients (92% sensitivity and 74% specificity). We also tested [15] the immune reaction against the N-terminal fragments of the GPI proteins agglutinin-like sequence 3 (Als3) and hyphal wall protein 1 (Hwp1), which showed lower specificity and accuracy values compared to our results for anti-Hyr1 and anti-D22b detection; however, the detection of anti- Als3 antibodies was more sensitive. It is worth mentioning that, in the present study, we used a subset of the sera tested in the previous study [15]. Interestingly, anti-Hyr1 and anti-D22b ELISA tests were more sensitive than CAGTA or BDG for the same group of patients. Although CAGTA titre determination showed the highest specificity and positive predictive values, close to 100%, it remains as a non-automatable and subjective technique that requires a prior treatment of the serum and operator training. In contrast, anti-Hyr1 or anti-D22b ELISAs do not have these limitations and provide diagnostic values very close to those of the CAGTA, including a higher sensitivity. In addition, the high negative predictive values for the detection of anti-Hyr1 (or D22b) antibodies could represent an accurate tool to discard IC in patients at risk, allowing the elimination of prophylaxis or de-escalation of empirical antifungal treatments, which in turn would reduce their side effects and the possible emergence of resistant strains and financial costs, and positively impacting antifungal stewardship.

The combinations of results proposed in this study improved the sensitivity compared to the individual tests. However, when any of the ELISA results were combined with those of the BDG, the specificity markedly decreased, as BDG is a panfungal biomarker. On the other hand, although good results were obtained with the combination of the developed ELISAs and the CAGTA technique results, this approach retains the limitations inherent to the immunofluorescence method. Finally, combining the two ELISA test results led to only a modest improvement, possibly because the antigens correspond to a protein and its fragment. Therefore, in future studies, it would be interesting to explore combinations of results of the developed ELISAs with those of independent biomarkers, such as antibodies against other CAGTA-recognized proteins, including Als3, Hwp1, and Met6.

Anti-Hyr1 antibodies were also detected in patients infected with non-*albicans Candida* species. This phenomenon of cross-reactivity with *C. albicans* specific proteins has already been described for CAGTA [16, 42]. Although no Hyr1 orthologs have been reported, the Iff/Hyr family has been identified in other species of the CTG *Candida* clade and is the most enriched gene family in pathogenic *Candida* isolates [43]. These Hyr-like family orthologs could explain the observed cross-reaction in non-*albicans Candida* species, as well as a lower sensitivity of the anti-D22b antibody assay for some species (*C. albicans*, *C.* *parapsilosis*), since homology within this protein family is concentrated in its N-terminal region [44], which is not included in the D22b fragment. Cross-reactivity with a monoclonal antibody against Hyr1 has also been reported for *Candida auris* (currently *Candidozyma auris*) [45], one of the species reported to have Iff/Hyr-like proteins [46]. Interestingly, *C. glabrata* infections were detected in the same proportion (81%) by the three antibody tests, anti-Hyr1, anti-D22b and CAGTA. These results are particularly noteworthy because they all pertain to hypha-specific antibodies and *C. glabrata* is not able to produce hyphae or pseudohyphae [47] and it does not belong to the CTG clade; however, a recent study [48] characterised a novel family of cell wall proteins in *C. glabrata* called Adhesin-like Wall Proteins (Awp). A member of this family, Awp2, which shares homology on its N-terminal region with the *C. albicans* Iff/Hyr family, has been implicated in the pathogenesis of the microorganism. The Awp2 protein, or one of the Awp family proteins that have yet to be characterised, may be responsible for the development of cross-reactive antibodies against Hyr1 and D22b. Further research is required to assess the utility of these biomarkers in cases of IC caused by other *Candida* species such as *Candida tropicalis, Candida guilliermondii* or *Candida krusei* as the small sample size for those species in this study does not allow clear conclusions.

In conclusion, the Hyr1 protein is subject to post-translational modifications that prevent its proper folding in a prokaryotic expression model such as *E. coli*. However, when produced in the yeast *P.* *pastoris*, a novel approach for this protein, it more closely resembles the native *Candida* protein and these modifications are essential for the accurate recognition of specific antibodies. The results of the detection of these antibodies, against the complete Hyr1 and D22b, a Hyr1-subterminal fragment, shows promising results for the early diagnosis of invasive candidiasis, including those caused by non-*albicans Candida* species. The ELISA assays overcome some of the drawbacks of blood culture, requiring only a few hours as opposed to 2–4 days for a positive blood culture. In addition, compared to the laborious immunofluorescence protocol required for CAGTA titration, ELISA is an objective and easily standardised technique not requiring serum pre-treatment, which makes it useful for monitoring patients at risk of invasive candidiasis and it is suitable for automation and large-scale application. Currently, several authors support biomarkers as an aid in the diagnosis of invasive candidiasis and the detection of anti-Hyr1 antibodies appears to be a good complementary test to blood culture.
